# RETRACTED: Albano et al. Systematic Review of Fatal Sodium Nitrite Ingestion Cases: Toxicological and Forensic Implications. *Toxics* 2024, *12*, 124

**DOI:** 10.3390/toxics12110819

**Published:** 2024-11-15

**Authors:** Giuseppe Davide Albano, Corinne La Spina, Roberto Buscemi, Mattia Palmeri, Giuliana Malandrino, Fausto Licciardello, Mauro Midiri, Antonina Argo, Stefania Zerbo

**Affiliations:** Section of Legal Medicine, Department of Health Promotion, Mother and Child Care, Internal Medicine and Medical Specialties, University of Palermo, 90129 Palermo, Italy

The journal retracts the article titled “Systematic Review of Fatal Sodium Nitrite Ingestion Cases: Toxicological and Forensic Implications” [1], cited above.

Following publication, concerns were brought to the attention of the Editorial Office regarding an overlap between this publication [1] and two previous publications [2,3] produced by different groups of authors.

Adhering to our complaints procedure, the Editorial Office and Editorial Board conducted an investigation that confirmed a significant overlap between [1] and two previous publications [2,3], without appropriate acknowledgment or citation. As a result, the Editorial Board has decided to retract this paper as per MDPI’s retraction policy (https://www.mdpi.com/ethics#_bookmark30).

This retraction was approved by the Editor-in-Chief of the *Toxics* journal. 

The authors did not agree to this retraction.
